# Differentiating pregnancies near the uterotubal junction (angular, cornual, and interstitial): a review and recommendations

**DOI:** 10.1186/s40738-020-00077-0

**Published:** 2020-05-04

**Authors:** Alex R. Finlinson, Kassie J. Bollig, Danny J. Schust

**Affiliations:** Department of Obstetrics, Gynecology and Women’s Health, MU Institute for Women’s Health Research, University of Missouri School of Medicine, 500 North Keene Street, Columbia, MO 65201 USA

**Keywords:** Eccentric pregnancy, Ectopic pregnancy, Angular pregnancy, Cornual pregnancy, Interstitial pregnancy, Tubal pregnancy, Uterotubal junction, Early pregnancy ultrasound

## Abstract

Eccentrically located intracavitary pregnancies, which include pregnancies traditionally termed as cornual and/or angular, have long presented complex diagnostic and management challenges given their inherent relationship to interstitial ectopic pregnancies. This review uses the existing literature to discriminate among interstitial, cornual, and angular pregnancies. Current arguments propose the outright abandonment of the terms cornual and angular may be justified in favor of the singular term, eccentric pregnancy. Disparate definitions and diagnostic approaches have compromised the literature’s ability to precisely describe prognosis and ideal management practices for each of these types of pregnancies. Standardizing the classification of these pregnancies near the uterotubal junction is important to unify conservative, yet safe and effective management strategies. We advocate the use of early first trimester ultrasound to correctly differentiate between eccentric pregnancy and interstitial ectopic pregnancy as current research suggests substantially better outcomes with correctly diagnosed and expectantly managed eccentric pregnancies than past investigations may have shown. The expectant management of eccentric pregnancies will often result in a healthy term pregnancy, while interstitial ectopic pregnancies inherently have a poor likelihood of progressing to viability. When the terms and diagnosis of cornual, angular, and interstitial pregnancy are indistinct, there is substantial risk of intrauterine pregnancies to be inappropriately managed as ectopic pregnancies. Until we standardize terms and criteria, it will remain difficult, if not impossible, to determine true risk for pregnancy loss, preterm labor, abnormal placentation, and uterine or uterotubal rupture. The development of best practice guidelines will require standardized terminology and diagnostic techniques.

## Introduction

Ectopic pregnancies account for only 2% of all reported pregnancies [1], but are responsible for 2.7% of pregnancy-related deaths and remain a leading cause of hemorrhage-related maternal mortality [2]. In most cases, differentiating intrauterine and extrauterine, or ectopic pregnancies, via state-of-the-art first trimester ultrasonography is fairly straightforward. Still, when the gestational sac is located eccentrically and near the uterotubal junction, such distinctions can be particularly challenging, and inaccurate localization can have substantial clinical ramifications. Inherent technological diagnostic challenges are amplified by an inconsistent and often confusing literature. Disparate definitions for pregnancies surrounding the uterotubal junction (angular, cornual, and interstitial pregnancies) have left a century old quandary concerning the differentiation of these clinical entities. As it stands today, definitive diagnosis of such pregnancies requires expedient standardization of terminology, treatment approaches, and counseling based on these standardized definitions. Such consensus is needed to optimize patient safety and maximize the future study of these conditions. This review aims to recommend standardized terminology that simplifies and distinguishes interstitial, cornual, and angular pregnancy and to provide insight into accurate methods for diagnosis and appropriate subsequent management of these clinical entities.

### Anatomy

The uterine cervix and corpus and the fallopian tubes arise from the Mullerian ducts with the former representing the fused portion and the latter the unfused portions of these embryologic structures [3]. The fallopian tube is divided into four segments. Beginning medially at the uterine junction and continuing laterally, these include the interstitial segment, the isthmus, the ampulla, and the infundibulum [3]. The interstitial segment is only one to two centimeters in length and is encompassed by a continuously thinning layer of uterine myometrium from its origin at the inner tubal ostium and traveling laterally to the isthmic portion of the fallopian tube [4]. The uterus is divided into two main regions with the superior two-thirds representing the corpus (body) of the uterus and the inferior one-third representing the cervix. The uterine cornua are the somewhat ill-defined, superolateral portions of the uterine body near the location of the internal tubal ostia and connecting fallopian tubes [4, 5]. The absolute boundary between the uterus and the fallopian tube, commonly referred to as the uterotubal junction, is a precise one, and can only be determined by pathologic examination that demarcates a change from uterine to fallopian tube cell types [6]. With reference to the uterine corpus, the term “angular” is even less well-defined. In fact, it does not refer to an anatomically distinct portion of the uterus, but instead is simply described as “relating” to the lateral angles of the uterine cavity [7]. Therefore, under strict anatomic definitions, the term cornual pregnancy should refer to an intracavitary gestation in the anatomic region of the uterine cornua, and the term interstitial pregnancy restricted to those extrauterine or ectopic pregnancies located within the interstitial portion of the fallopian tube. The less well-defined term angular pregnancy, if used, might then include all laterally and superiorly placed intrauterine gestations, including those defined as cornual above. Such overlapping definitions are often not particularly useful from a diagnostic or therapeutic standpoint. Further complicating these definitions is the fact that the interstitial portion of the fallopian tube is technically within the uterus, albeit within the fallopian tube as it passes through the uterine myometrium. Therefore, while not intracavitary, it is intrauterine (see below). We will address, in turn, each of the terms angular, cornual, and interstitial as they refer to pregnancies with a goal of coalescing the literature and developing more useful terminology that reflects prognosis and management.

### Definitions and diagnoses

#### Angular pregnancy

Ill-defined and inconsistent use of the term “angular pregnancy” dates back to its first use by Dr. Howard Kelly well over a century ago. In 1898, he first defined an angular pregnancy as, “implantation of the embryo just medial to the uterotubal junction, in the lateral angle of the uterine cavity.” [8] It was nearly 100 years before the terminology was next addressed in a systematic fashion. In 1981, Janson and Elliot observed that the terms angular and interstitial were being used synonymously for two very different types of laterally displaced or eccentric pregnancies [7]. In an attempt to clarify the differences between these two types of gestations, they employed the use of laparoscopy to describe these pregnancies in relationship to the round ligament stating, “The lateral uterine enlargement of an angular pregnancy displaces the round ligament reflection upward and outward. The swelling of an interstitial tubal pregnancy is lateral to the round ligament.” [7] This more recent distinction and set of criteria has persisted even to publications within the past few years, with the diagnosis of angular pregnancy defined by: 1) clinical presentation with painful asymmetric enlargement of the uterus, 2) directly observed (i.e., surgical) lateral distension of the uterus with displacement of the round ligament laterally, and 3) retention of the placenta in the uterine angle [7].

#### Cornual pregnancy

The earliest descriptions of “cornual pregnancy” date back to 1952, when Johnston and Moir [9] clearly ascribed the term to pregnancies located “in one horn of a bicornuate uterus, or, by extension of meaning, in one lateral half of a uterus of bifid tendency.” Concordant with this earliest definition, most groups reporting cases and outcomes of cornual pregnancy have attempted to limit the specific term “cornual pregnancy” to **intrauterine** implantations in an anomalous unicornuate, bicornuate, or septate uterus (Fig. 1) [10–13]. A smaller proportion of scholars, however, support a more anatomic interpretation to define cornual pregnancies and this inconsistency confuses the literature. As soon as 20 years after the first definition by Johnston and Moir, Maher and Grimwade argued that many providers labeled a pregnancy as cornual if located in the cornua of the uterus, whether it was in an anomalous uterus or normal uterus. They wrote, “the fact remains that any pregnancy occurring in the cornual region of a normal uterus is still referred by many gynecologists as a ‘cornual pregnancy’.” [14] In agreement with this sentiment, older versions of *Williams Obstetrics* used Maher and Grimwade’s definition of the term. However, with increasing support and endorsement by high impact OB/GYN journals, the most current version of *Williams* defines cornual pregnancy as “a conception that develops in the rudimentary horn of a uterus with a mullerian anomaly.” [10, 11] Still, it is not uncommon in general practice to hear radiologists, ultrasonographers and practitioners caring for reproductive-aged women to use the term cornual pregnancy to refer to a range of gestations that occur near the uterine cornua, including intracavitary and ectopic pregnancies in normal and anomalous uteri.

**Image 1:**
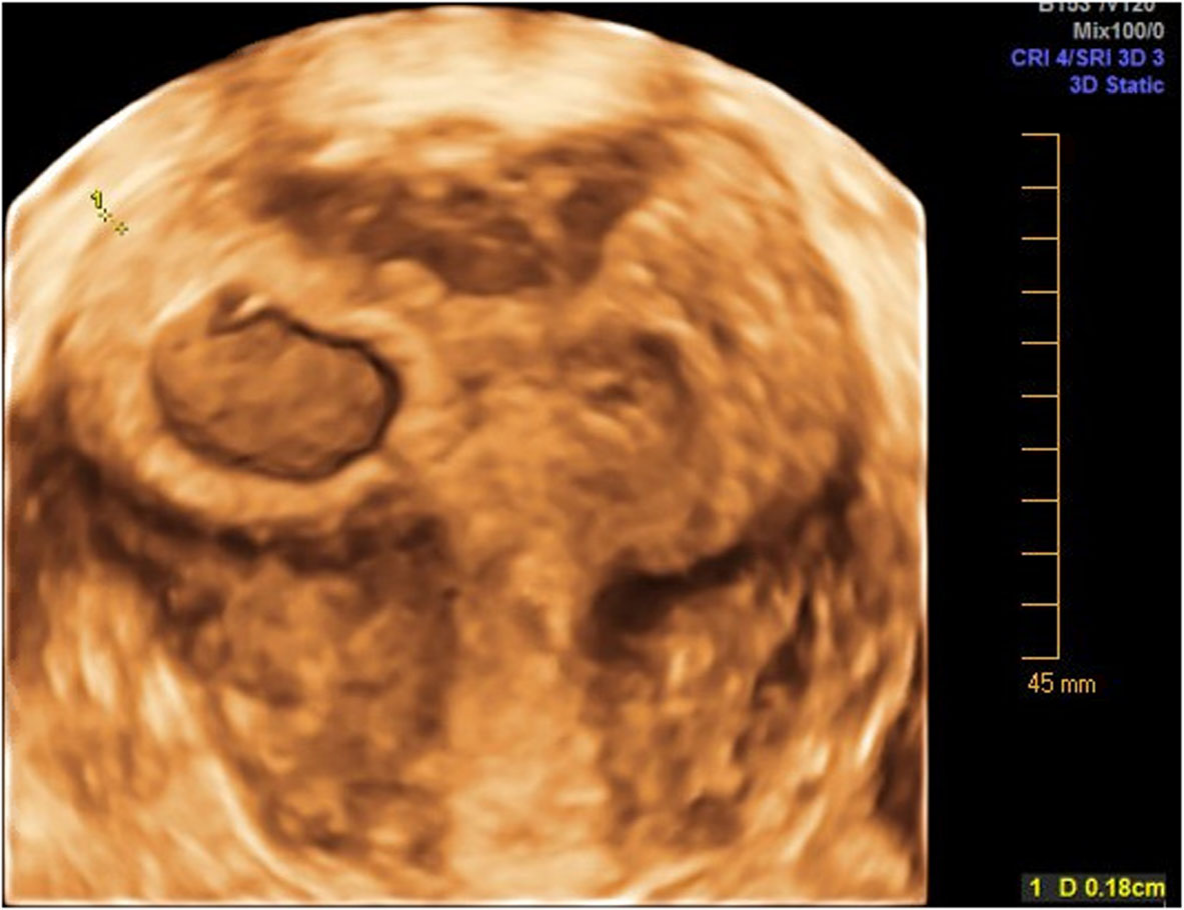
Eccentric pregnancy in a partially septate uterus, which previous literature referenced as a “cornual pregnancy”

#### Interstitial pregnancy

Least controversial is the term “interstitial ectopic pregnancy”, one which we would pose should remain in the lexicon. Although it is common to hear that ectopic pregnancies are those that are outside of the uterus, the increase in cervical ectopic pregnancies and cesarean scar pregnancies make evident that this description is imprecise. Written literature and standard textbooks such as *Williams Obstetrics*, however, are more clear and consistent in their definition of ectopic pregnancies as gestations that have implanted outside the **endometrial cavity** [10]. Ectopic pregnancy classically presents with any combination of a missed period, unilateral lower abdominal pain, and vaginal bleeding or spotting. Ectopic pregnancies can occur in a range of places, including the fallopian tube, ovary, uterine cervix, cesarean section scars, intestine, or other intra-abdominal locations. The fallopian tube is the most common site of implantation outside of the endometrial cavity, occurring in 90% of cases [15]. Interstitial tubal pregnancies are the least common of the tubal ectopic gestations [16] and are estimated to account for 2–4% of ectopic pregnancies overall [17]. They have a rupture rate of 13.6%, a maternal mortality rate of 2–2.5%, and have been quoted as accounting for 20% of all deaths attributed to ectopic pregnancy [7, 18, 19]. The term “interstitial pregnancy” is also fairly consistently and clearly defined as a gestation that implants within the proximal tubal segment that lies within the muscular uterine wall [8]. While these types of pregnancies could certainly still be considered within the uterus, like cervical and cesarean scar pregnancies, they lie outside of the endometrial cavity.

Ultrasound criteria that attempted to accurately diagnose the interstitial ectopic pregnancy were first proposed in the early 1990s by Timor-Tritsch and include: 1) an empty uterine cavity, 2) a chorionic sac [seen] separately (1 cm) from the lateral edge of the uterine cavity, and 3) a thin (5 mm) myometrial layer surrounding the chorionic sac [20]. These criteria were shown to accurately differentiate interstitial ectopic pregnancies from other types of pregnancies with relatively high (90%) specificity. Shortly thereafter, others suggested the addition of an “interstitial line sign” to the above criteria [21]. The interstitial line sign is an echogenic line seen by transvaginal sonography in the cornual region of the uterus bordering the midportion of the gestational sac and connecting it to the endometrial cavity proper. The interstitial line sign is thought to represent the interstitial portion of the fallopian tube proximal to the ectopic gestation. Adding this new criterium to those of Timor-Tritsch, Grant and associates reported a diagnostic sensitivity of 80% and specificity of 90% [22].

In 2017, Grant and associates studied an alternative approach to discriminating between intrauterine but eccentrically located pregnancies and interstitial ectopic pregnancies by proposing application of a “double sac sign” to characterize the eccentric but intrauterine pregnancy [22]. This sign was first described in the 1980s as a means to sonographically verify all intrauterine pregnancies and uses the presence of two concentric, sonolucent intrauterine rings surrounding the gestational sac to determine an early intrauterine pregnancy [23]. These rings are thought to represent an outer, normal peripheral decidual reaction and an inner chorionic ring. These authors reported that half of eccentric pregnancies were potentially misidentified as interstitial ectopic pregnancies and that use of the “double sac sign” to reliably distinguish “surrounding endometrium” discriminated between eccentric intrauterine and interstitial ectopic pregnancies with promising interobserver and intraobserver agreement (kappa 0.91 and 0.95, respectively) and a specificity of 100% [22]. Historical definitions of each of these three entities are summarized in Table 1.

**Table 1:** Common definitions of pregnancy near the uterotubal junction in the literature

**Ectopic Pregnancy** Pregnancy outside the endometrial cavity	
**Interstitial Pregnancy** - Pregnancy that implants within the proximal tubal segment that lies within the muscular uterine wall [10]. - Implantation within the most medial 1–2 cm of a fallopian tube as it opens into a uterine cavity without evidence of uterine anomaly [24].	
**Eccentric Pregnancy** Pregnancy implantation within the superior-lateral aspect of the endometrial cavity/uterine corpus	
**Angular Pregnancy**: - Implantation within the endometrial cavity, but at one cornua and medial to the uterotubal junction and round ligament [24]. - Angular pregnancy that displaces the round ligament upward and outward, whereas interstitial tubal pregnancy will not [12]. - Lateral displacement of pregnancy by a uterine leiomyoma or other myometrial mass [25].	
**Cornual Pregnancy:** - Conception that develops in the rudimentary horn of a uterus with a mullerian anomaly [11].	

### Prognosis & Management

The most striking reason for standardizing the classification of the several types of pregnancies that can be found near the uterotubal junction lies in the markedly distinct prognoses and management approaches of the different conditions. There were over 100 years separating the first description of angular gestations by Howard Kelly in 1898 and a subsequent historical case analysis published in 2014 [7, 26]. Using Jansen and Elliot’s diagnostic criteria, much of the clinical data collected over an approximately 80 year period involved symptomatic patients diagnosed in the second trimester. For these patients, it was reported that spontaneous abortion rates for angular pregnancies ranged from 18% to 38.5% and uterine rupture rates from 13.6% to 28% [7, 26]. Management of these pregnancies overwhelmingly involved surgical intervention in the form of diagnostic laparoscopy at minimum [3], and, more commonly, pregnancy termination [7, 26]. In fact, some recent research has been performed to determine the most efficient and safest method for termination of angular, cornual, and interstitial pregnancies [27–32]. In contrast, several investigators have recently argued for expectant management of eccentric intrauterine pregnancies. In a 2017 publication, Grant, et al., reported no uterine ruptures in a fairly small retrospective review of cases diagnosed in the first trimester among patients seen in three tertiary care institutions in Canada [22]. While it is difficult to determine the true spontaneous abortion rate in these patients because many had active intervention rather than expectant management, it is important to note that on retrospective review, 10 potential eccentric intrauterine pregnancies were managed as interstitial ectopic pregnancies. In other words, 10 potentially viable pregnancies were terminated [22]. While the sample size was small in this study, it highlights the importance of establishing consistent definitions for and outcomes of eccentric pregnancies [33].

One recent review of eccentric pregnancy case reports demonstrated a large range for live birth rates (25–69%) [26] when utilizing specific sonographic criteria to diagnose eccentric pregnancy in the first trimester. Recently published data from the University of Missouri indicates even more reassuring outcomes with conservative management of eccentric pregnancies. In the latter study, 80% of cases resulted in a live birth [13] and there were no cases of uterine rupture, maternal death, abnormal placentation, or hysterectomy. We would argue that if intrauterine pregnancies implanted near the uterotubal junctions of normal **and** anomalous uteri are grouped under the term “eccentric” pregnancies and diagnosis is made in the first trimester, management should nearly always be expectant since pregnancy outcomes are often quite good [13]. Complications rarely include those that would jeopardize maternal health and most commonly occur in the third trimester as premature labor and fetal malposition (the latter particularly for some congenital uterine anomalies). Management of an eccentric pregnancy diagnosed in the second trimester and associated with maternal pain is more controversial and at this time would require more personalized care based on the clinical scenario [22, 34].

In stark contrast, interstitial ectopic pregnancies have a vanishingly small chance of progressing to viability. A literature review in 2013 reported a total of 11 live births after diagnosis of interstitial ectopic pregnancy, although all were associated with significant morbidity, including uterine rupture, placenta accreta spectrum disorders, and fetal growth restriction [35]. Based on the available literature, intervention is warranted at the time of diagnosis of interstitial ectopic pregnancy to possibly allow for less invasive therapeutic approaches that minimize maternal morbidity. The case mortality rate for interstitial ectopic pregnancy is quoted to be 2–3%, a mortality rate two fold greater than most other forms of ectopic pregnancy [36, 37]. Definitive surgical management of interstitial ectopic pregnancies has included cornual resection, cornuostomy, salpingostomy, hysteroscopic injection, hysteroscopic removal of the gestation, and even hysterectomy [17, 38, 39]. While the diagnosis of interstitial ectopic pregnancy has historically called for surgical management, ultrasound guided injections and medical management with methotrexate are becoming increasingly common choices when diagnosis is made early [40, 41]. Medical management with methotrexate has an overall success rate of 83% when using local, systemic, or combined administrations [17]. Other isolated cases in the literature have used local administration of potassium chloride or actinomycin D rather than targeted or systemic methotrexate [42–44].

### Recommendations

The lack of clarity in terminology used to define the several types of pregnancies that localize near the uterine cornua can have dramatic consequences. If the terms cornual, angular, and interstitial pregnancies are used interchangeably, there is substantial risk for intrauterine pregnancies to be inappropriately managed as ectopic pregnancies and vice versa. Imprecise definitions and inconsistent use and timing of diagnostic modalities have clouded the literature’s ability to estimate prognosis of and best practices for management of each of these types of pregnancies. Until we standardize diagnostic criteria, it will remain difficult, if not impossible, to determine true risk for pregnancy loss, preterm labor, abnormal placentation, and uterine or uterotubal rupture [7, 22, 26].

We should use the current environment of increasingly skilled providers and advancing technology to aid standardization efforts. The risks of relying on past definitions despite improved diagnostic capabilities are perhaps most evident when examining the widespread continued use of Jansen and Elliot’s criteria to diagnose angular pregnancy. This set of criteria relies on a surgical procedure (diagnostic laparoscopy) or a patient’s clinical symptoms, typically in the midtrimester, for definitive diagnosis. In 2014, utilizing these criteria, Arleo and Defilippis found less than 100 reported cases of this type of eccentric pregnancy in the literature and most were diagnosed in the second trimester of pregnancy when clinical symptoms warranted surgical intervention [26]. The authors stated in their review that although “there is no absolute anatomic boundary distinguishing an angular pregnancy from a normal one, the closer a gestation implants to the internal uterine ostium of the fallopian tube, the greater likelihood of visual asymmetry and a symptomatic patient as the pregnancy progresses.” [12] While technically true, our own experience [13] suggests that this may be less problematic when transvaginal two- and three-dimensional scanning is performed early in pregnancy. Rapid expansion of first trimester transvaginal sonography has forced practitioners to more commonly confront laterally placed gestations much earlier in pregnancy than what has been historically reported. A sonographic diagnosis of an eccentric intrauterine pregnancy in the first trimester in an asymptomatic patient may have very different clinical implications from diagnosis later in pregnancy in the presence of symptoms [13]. While early sonographic assessments are rapidly becoming common practice in many general obstetrics practices for both asymptomatic and symptomatic pregnancies, they are almost universal in infertility practices, where the risk of ectopic gestation is amplified by assisted reproduction [25, 45].

Existing circular and ultimately confusing arguments suggest that abandonment of the terms cornual and angular may be justified in favor of the singular term, **eccentric pregnancy**, that describes a pregnancy within the endometrial cavity that has implanted near but medial to the uterotubal junction. It remains unclear whether using separate terms to discriminate eccentric pregnancies in non-anomalous versus anomalous uteri has prognostic utility, particularly when diagnosis is made in the first trimester [13]. Simplifying and standardizing terminology would certainly improve our ability to better study this question. At a minimum, angular and cornual pregnancies should be recognized as intrauterine pregnancies and use of terminology that impairs differentiation from ectopic pregnancies, specifically interstitial ectopic pregnancies, should be discontinued [13, 22].

Already, groups of investigators have started to develop and utilize newer ultrasound “signs” and criteria that can make early detection of these pregnancies possible and prevent potentially dangerous patient outcomes [17, 22, 34]. Continued use and validation of these ultrasound techniques will add to overall knowledge and insight into the natural history of eccentric intrauterine and interstitial ectopic pregnancies. Furthermore, retrospective and prospective studies utilizing modern instrumentation and consistent ultrasound criteria will help to both guide and better reflect current practice in early pregnancy. Using additional data based on current practice and consistent terminology, newly developed practice guidelines can more accurately define prognosis and management.

## Conclusion

The terms angular and cornual pregnancy have been used to describe pregnancies located near the uterine cornua, but still within the uterine or endometrial cavity. For the sake of anatomical and diagnostic simplicity, we advocate both terms should be abandoned. Instead, use of the more inclusive and encompassing term, eccentric pregnancy could simplify distinction from the more morbid interstitial ectopic pregnancy. Consistent and dependable diagnosis of eccentric intrauterine and interstitial ectopic pregnancies remains a challenge, but has and will continue to improve with the advancement of ultrasound technology. There remains significant opportunity to formulate and disseminate criteria that discriminate between and classify pregnancies that are only millimeters apart, but have disparate outcomes that range from the delivery of a healthy full-term infant to pregnancy loss and potential maternal morbidity. Current literature provides sonographic criteria (Tables 2 and 3) that could be standardized and implemented broadly for the diagnosis of eccentric pregnancy [8, 13, 21, 22]. The importance of reliably distinguishing between eccentric and interstitial ectopic pregnancies cannot be overstated. The actual prevalence of such pregnancies, their clinical course, and associated risks can only be adequately defined though further research. Ultimately, accurate characterization of these pregnancies will elevate and guide more efficient delivery of care for pregnancies localized near the uterotubal junction.

**Table 2:** Sonographic criteria for eccentric pregnancy

**Eccentric Pregnancy Ultrasound Criteria:**	
**1.** Implantation of the embryo in the lateral angle of the uterine, cavity, just medial to the uterotubal junction [8]. **2.** ≤ 1 cm of myometrial thickness surrounding the gestational sac [22]. **3.** Presence of completely circumferential endometrium surrounding the gestation, and therefore diagnostic of intrauterine gestation. “Surrounding endometrium” or “double sac sign” [22] **4.** Lack of an “interstitial line sign” or extension of endometrium to the gestational sac edge [21].

**Table 3:** Sonographic criteria for interstitial pregnancy

**Interstitial Pregnancy Ultrasound Criteria:**	
**1.** An empty uterine cavity [20]. **2.** A chorionic sac identified separately from the lateral edge of the uterine cavity by at least 1 cm [20]. **3.** A thin, 5 mm myometrial layer surrounding the chorionic sac [20]. **4.** Presence of an “interstitial line sign” or extension of endometrium to the gestational sac edge [21].	

## Data Availability

Not Applicable.
